# Development of a colloidal gold-based immunochromatographic strip for rapid detection of *Areca palm velarivirus 1*

**DOI:** 10.3389/fmicb.2025.1533170

**Published:** 2025-02-20

**Authors:** Jie Lu, Weifan He, Yuxing Liu, Shiqi Li, Xi Huang, Hongxing Wang, Xianmei Cao

**Affiliations:** School of Breeding and Multiplication (Sanya Institute of Breeding and Multiplication), Hainan University, Haikou, China

**Keywords:** *Areca palm velarivirus 1* (APV1), monoclonal antibodies (MAbs), colloidal gold immunochromatographic strip, rapid detection, surveillance

## Abstract

**Background:**

*Areca palm velarivirus 1* (APV1), the causal agent of betel palm yellow leaf disease (YLD), has caused significant yield losses and decreased product quality of betel nuts, posing a serious economic threat to local farmers. There is an urgent need for a convenient and reliable method for the rapid detection and surveillance of APV1.

**Methods:**

The Capsid protein (CP) of APV1 was expressed in *Escherichia coli* and purified as antigen to immunize BALB/c mice. Two specific monoclonal antibodies (MAbs), APV1CP-1 and APV1CP-10, were generated through the hybridoma technique. APV1CP-1 was conjugated with colloidal gold served as detection reagent, while APV1CP-10 was coated onto a porous nitrocellulose membrane to act as the detection line. Goat anti-mouse IgG was used as the control line. These components were then assembled into a colloidal gold immunochromatographic strip (CGICS) for effective detection of APV1.

**Results:**

The MAbs APV1CP-1 and APV1CP-10 were successfully obtained with titers exceeding 1:102,400. Colloidal gold particles used in the assay had an approximate diameter of 30–40 nm, and exhibited a surface plasmon resonance peak around 530 nm. The CGICS allowed for the detection of APV1 by applying infected sap to the test strip, with results visible within 5–10 min. The test showed no cross-reactivity with other viruses tested, and the visual detection limit for APV1 was established at a 100-fold dilutions of APV1-infected leaf samples.

**Conclusion:**

The monoclonal antibody-based colloidal gold immunochromatographic strip developed in this study demonstrates significant convenience, rapidity, and reliability for APV1 detection. These advancements are anticipated to facilitate rapid diagnosis and surveillance of APV1 in field settings.

## 1 Introduction

Areca palm (*Areca catechu* L.) is a versatile perennial evergreen and a valuable economic crop in Southeast Asia (Khan et al., 2023). In China, Hainan Province is the principal production region for areca palms. In 2020, the total output value of areca nut fruits in Hainan reached 14.68 billion yuan (approximately 2 billion USD), making it a crucial income source for 2.2 million farmers and accounting for around 40% of the agricultural population in the province (Cao et al., 2024). As areca palm plantations expand, they face threats from various pathogens, including bacteria, fungi, insects (such as *Tirathaba rufivena* and *Brontispa longissima*), phytoplasmas, and viruses (Ramaswamy et al., 2013; Khan et al., 2022). YLD is one of the most severe diseases affecting areca palm, occurring across all major production areas worldwide (Nair et al., 2016; Wang et al., 2020). Recent studies have identified APV1 in YLD samples through *de novo* assembly, RNA-sequencing, and next-generation sequencing (Yu et al., 2015). Comparative analysis of symptomatic vs. symptomless tree populations has demonstrated a strong association between APV1 and YLD (Wang et al., 2020). Furthermore, based on the experiment of insect-borne inoculation, *Pseudococcus cryptus* and *Ferrisia virgata* were identified as the responsible transmission vectors of APV1 in Hainan (Zhang et al., 2022; Zhao et al., 2024), thereby causing typical YLD symptoms in areca palm seedlings. Additionally, viral titers of APV1 are higher in winter compared to summer, as shown by quantitative RT-PCR (qRT-PCR) and ELISA assays, and YLD-affected areca palms exhibit more severe yellowing symptoms during winter based on field observations (Khan et al., 2022).

APV1 belongs to genus *Velarivirus* within the family *Closteroviridae*. It is characterized by its typical flexuous, filamentous viral particle and a long, positive-sense, single-stranded RNA genome that encodes 11 open reading frames (ORFs). ORF1a and ORF1b encode a large protein with replication-related properties. ORF2 encodes a 4-kDa hydrophobic protein. ORF3 encodes a 70-kDa heat-shock protein 70 homolog (HSP70h), which partially overlaps ORF4, encoding a 21-kDa polypeptide. ORF5 encodes a 60-kDa protein. ORF6 and ORF7 encode the coat protein (CP) and CP minor (CPm), respectively. ORF8, ORF9, and ORF10 encode polypeptides with unknown functions (Wang et al., 2020). Phylogenetic analysis has classified APV1 isolates into three distinct phylogroups. Phylogroup A is identified as the most prevalent APV1 genotype in areca palm plantations in Hainan, China (Cao et al., 2021). Currently, genetic engineering techniques are considered the most effective method for controlling plant virus diseases (Abel et al., 1986; Carbonell and Daros, 2019). However, the effectiveness of the genetic engineering techniques mentioned above is limited by the lack of efficient genetic transformation methods for areca palm (Khan et al., 2023). As a result, there is no efficacious approach to eradicate viral diseases in this crop. Therefore, developing rapid and efficient detection technology is crucial for identifying infected seedlings, monitoring the progression of the disease, and controlling the spread of APV1. Several detection techniques, including reverse transcription-polymerase chain reaction (RT-PCR) (Cao et al., 2021), quantitative RT-PCR (qRT-PCR) (Lin et al., 2021; Khan et al., 2022), enzyme-linked immunosorbent assay (ELISA) (Chen et al., 2022), multiplex PCR (Peng et al., 2023), and high-throughput sequencing, have been employed to identify APV1 in areca palm (Cao et al., 2021). However, these traditional detection methods are often time-consuming, laborious, and expensive, and they require specialized laboratory equipment, making them impractical for large-scale field surveys. In contrast, the colloidal gold immunochromatography assay (CGICA) offers a rapid detection solution by combining the specificity of antigen-antibody interactions with colloidal gold labeling and immunochromatography. CGICS have been successfully employed for the diagnosis of various plant viruses, such as *Citrus tristeza virus* (CTV) (Salomone et al., 2004), *Lily mottle virus* (LMoV) (Zhang et al., 2015), *Tomato zonate spot tospovirus* (TZSV) (Niu et al., 2018), *Rice stripe virus* (RSV) (Huang et al., 2019), *Soybean mosaic virus* (SMV) (Ren et al., 2022), *Banana bract mosaic virus* (BBrMV) (Selvarajan et al., 2020), and so on. Despite its successes with other plant viruses, there is currently no systematical report on the application of CGICS for diagnosing APV1 infection. CP and CPm encapsulate approximately 95% of the 3′ terminus and the terminal 5% region of the filamentous viral genome RNA (gRNA), respectively. These proteins are indispensable for the virus to successfully infect its host. The CP, in particular, stands out as a structurally robust and highly specific constituent of the virus particle. It not only effectively embodies overall traits of the virus, but also owing to its surface-exposed location, readily allows for antibody recognition. This feature substantially augments the sensitivity and specificity of detection assays designed to identify the virus, providing a more reliable means of diagnosis (Dolja et al., 2006; Weber and Bujarski, 2015).

In this study, purified recombinant APV1-CP protein was obtained and used to develop specific MAbs APV1CP-1 and APV1CP-10. These MAbs were then utilized to create a novel, sensitive, and user-friendly CGICS for detecting APV1. The test strip provides results within 5–10 min and specifically detects APV1 without cross-reacting with other unrelated plant viruses. Additionally, the strip meets the sensitivity requirements for effective detection. This approach shows promising potential for preventing the spread of APV1.

## 2 Materials and methods

### 2.1 Plant materials

Samples were collected from areca palm plantations in Yazhou District, Sanya City, Hainan Province. Areca palm seedlings were intentionally infected with APV1 via inoculation by *Ferrisia virgata* (Zhang et al., 2022), resulting in observable yellowing symptoms. These samples were then transferred to the laboratory for further study. Areca palm infected with *areca palm necrotic ringspot virus* (ANRSV) and *areca palm necrotic spindle-spot virus* (ANSSV) were sourced from areca plantations in Yazhou District, Sanya City, Hainan Province.

### 2.2 Prokaryotic expression and purification of APV1-CP recombinant protein

Protein induction and purification experiments were carried out as previously described (Chen et al., 2022). Briefly, the coding sequences of CP was amplified and inserted into the pET-30a vector, incorporating the appropriate 6xHis tags. These constructs were then transformed into *Escherichia coli* BL21 (DE3) strain (WEIDI, EC2200S, Shanghai, China). The His-tagged soluble proteins were purified from the supernatant using BeyoGold His-tag Purification Resin (Beyotime, P2210, Shanghai, China) following the manufacturer’s instructions. The purified proteins were analyzed by SDS-PAGE.

### 2.3 Preparation and identification of MAbs against APV1-CP

Antibodies were prepared by Beijing Huada Protein R & D Center Co., Ltd. (Beijing, China), following the method as described (Niu et al., 2018). To produce APV1-specific MAbs, five eight-week-old female BALB/c mice were intraperitoneally and subcutaneously immunized with purified His-CP. The purified recombinant His-CP was mixed with an equal volume of Freund’s complete adjuvant (BioFroxx, CAS: 2203ML010, Germany) by repeated stirring to prepare the water-in-oil emulsion. The emulsion, containing 200 μg of antigen protein per mouse, was injected into the peritoneal cavity of 8-week-old BALB/c female mice. Fifteen days later, the purified recombinant His-CP was mixed with an equal volume of incomplete Freund’s adjuvant (BioFroxx, CAS: 1643ML010, Germany) and administered to the mice every 14 days for a total of three injections. Seven days after the last immunization, antiserum samples were obtained from the tail vein of each mouse. The resulting antisera were assessed for titers against serial dilutions of the corresponding antigen (APV1) using indirect ELISA, and the specificity of antibody serum to the immune antigen was determined by western blot.

### 2.4 Western blot and ELISA analysis of MAbs

Total proteins were isolated and separated by SDS-PAGE. The proteins were transferred from the gel onto a PVDF membrane (Millipore, CAS: IPVH00010, United States) using a Mini Trans-Blot Electrophoretic Transfer system (BioRad, #1703930). The PVDF membrane was then blocked with 10 mL of 5% milk (BD Difco, CAS: 232100, United States) in PBST [PBS containing 0.05% (v/v) Tween-20 (Biofroxx, CAS: 9005-64-5, Germany), pH 7.4] at 37°C for 1 h. Next, 10 mL of antiserum diluted with 5% milk in PBST (1:5,000) was added to cover the PVDF membrane, which was incubated at 4°C overnight with anti-APV1 MAbs. After three washes, horseradish peroxidase-conjugated goat anti-mouse IgG (diluted to 1:2,000) as the secondary antibody (Solarbio, Catalog No. SE131), was applied to the PVDF membrane and incubated at 37°C for 1 h. The blot was visualized using SuperSignal^™^ West Pico PLUS Chemiluminescent Substrate (Thermo Fisher, Catalog No. 34577).

ELISA analysis was conducted as previously described (Khan et al., 2022). The 96-well microliter plate (Nest Scientific, United States) was first coated with antigen (100 μL/ well) and was incubated at 4°C, overnight. After washing with PBST and blocking with 2.5% skimmed milk (200 μL/well), the primary antibody (100 μL/well) MAb was diluted at a ratio of 1:5,000 in 1× PBST buffer, was added to each well, and was then incubated at 37°C for 2 h. After washing with PBST, alkaline phosphatase-labeled goat anti-mouse IgG secondary antibody (100 μL/well) diluted with 1× PBST buffer at a ratio of 1:10,000 was added to each well and incubated at 37°C for 2 h. After that, the freshly prepared substrate tetra-methyl benzidine (TMB) solution (Solarbio, Cat#PR1200) (100 μL) was added to each well and was kept in the dark for 30 min at 37°C until the color developed to the desired level. The reaction was stopped by adding a stopping solution of 1 M concentrated HCl (Aladdin, CAS: 7647-01-0, Shanghai) (50 μL/well), and the reading was taken at OD 450 nm (BioTek Synergy H1, Microplate Reader, United States).

### 2.5 Preparation, purification and isotyping of immunoglobulin

The mouse showing the highest titer of the antiserum was selected for preparing spleen lymphocytes, which were then fused with SP2/0 myeloma cells. Screening was performed 10 days after fusion, and positive clones were selected out by indirect ELISA with the supernatants of clones after one week to ten days culture. With the secondary screening, positive clones were ultimately selected by indirect ELISA. The selected positive hybridoma cell lines were subsequently sub-cloned by injecting into the BALB/c mice. The ascites fluid of the sub-cloned BALB/c mice was collected, and the immunoglobulin class and subclass of the hybridoma lines were determined. Ultimately, the monoclonal antibody was purified.

### 2.6 Preparation of colloidal gold-conjugated antibody

To prepare the colloidal gold solution, 100 mL of 0.01% chlorauric acid solution (Sigma-Aldish CAS: G4022) was placed on a magnetic stirrer (IKA RET basic, Germany) and agitated at room temperature at 500 rpm for 10 min. Then stir while heating to boiling, and then immediately add 1 mL 1% trisodium citrate solution (Sangon Biotech, CAS: 6132-04-3), continue heating while stirring for 10 min, until the color of the colloidal gold solution changes from light yellow to dark red, stop heating, stirring for 10 min. After cooling the colloidal gold solution to ambient temperature, it is filled to the original volume with deionized water. The solution bottle is wrapped in tin foil to avoid light, and stored at 4°C for future use. The absorption maxima (max) of the solutions were analyzed using ultraviolet/visible spectroscopy (UV/Vis) with a Biotek Synergy H1 (Vermont, United States) to determine the approximate particle sizes, which were confirmed by transmission electron microscopy (TEM) measurements with a JEM-F200 transmission electron microscope (JEOL, Tokyo, Japan). Determination of optimal pH for Colloidal Gold-Labeled Antibody were performed as previously described (Wan et al., 2022; Sun et al., 2023). 0.2 mol/L K₂CO₃ solution (Sangon Biotech, CAS: 584-08-7) was used to adjust the pH of five tubes of colloidal gold solution to 6.0–10.0, with each tube containing 1 mL. In each pH-adjusted solution, 12.5 μL of APV1CP-1 (1.5 mg/mL) was added to achieve a final concentration of 12 μg/mL. The mixtures were incubated at room temperature for 2 h and then centrifuged at 12,000 rpm for 30 min at 4°C. The supernatants were collected, and the OD₅₃₀ values for each pH gradient were measured using a spectrophotometer.

To determine the optimal amount of the MAb APV1CP-1 for conjugation with the colloidal gold solution, colloidal gold (125 μL) was mixed with a series of gradient dilutions of the purified monoclonal antibody APV1CP-1, with each gradient supplemented with 125 μL of 10% NaCl. As the antibody proportion increased, the color of the colloidal gold solution shifted from light to deep purple. The gradient at which the color stabilized indicated the optimal antibody concentration.

To obtain the colloidal gold labeled antibody complex, 10 mL of colloidal gold solution was poured in a 50 mL conical bottle on a magnetic agitator, and the pH was adjusted to 8.0 with 0.2 M K_2_CO_3_ solution (pH was determined using pH indicator strips). Forty microliters of MAb were added to the colloidal gold solution at a ratio of 250:1 and stirred for 20 min. Then, 500 μL PEG20000 (20 mg/mL) (Sangon Biotech, CAS: 25322-68-3) was added and mixed for 20 min. Centrifuge 10,000 g at 4°C for 30 min and discard the supernatant. The colloidal gold-labeled antibody complex was generated through re-suspension precipitation with 500 μL pH7.4 PBS buffer containing 2%BSA (Biofroxx, CAS: 9048-46-8, Germany) and kept at 4°C for further use.

### 2.7 Assembly of the CGICS

The CGICS was composed of a MAb-gold conjugated pad (JINBIAO BIO, CAS: RB65, Shanghai), a sample pad (JINBIAO BIO, CAS: RB65, Shanghai), an absorbent pad (JINBIAO BIO, CAS: CH37, Shanghai) and an NC membrane (Huamike CN140, Beijing). The pads and the NC membrane were all pasted onto an adhesive plastic backing. The NC membrane was pasted at the center of the backing plate. The MAb-gold or PAb-gold conjugate pad was pasted by overlapping 1 mm on the bottom of the NC membrane, the sample pad was pasted by overlapping 2 mm on the bottom of the MAb-gold or PAb-gold conjugate pad and the absorbent pad was pasted by overlapping 1 mm on the upper position of the NC membrane. The whole assembled one-step strip was cut lengthways into 3.00-mm-wide strips using the guillotine cutter (Niu et al., 2018; Selvarajan et al., 2020).

### 2.8 Test procedure of the immunochromatographic strips

Approximately 200 mg of areca palm leaves was cut into thin strips and put into a mortar. After adding 2 mL of 0.01 mol/L PBS buffer (pH10) and silica sand (Guangzhou Chemical Reagent Factory, CAS: 14808-60-7, Guangzhou), the leaves strips were ground with grinding rod thoroughly, the crude extract was filtered using a syringe with 0.45 μm filter (BKMAMLAB, CAS: 110414006, Hunan). Approximately 150 μL of the filtered fluid (2–3 drops) were dropped individually into the sample pad. After 5–10 min, the samples showing two purple bands at the T and C lines were considered as APV1 positive, while the samples showing only one purple bands at the C line were considered as APV1 negative. If the C line did not show a purple color band, the test was considered as invalid.

### 2.9 Detection of APV1 infection through RT-PCR

Briefly, total RNA was extracted using the RNAprep Pure Plant Plus Kit (TIANGEN BIOTECH, China) according to the manufacturer’s instructions. cDNA was synthesized using random hexamer primers and the RevertAid First Strand cDNA Synthesis Kit (Thermo Fisher Scientific), following the manufacturer’s instructions. The conserved primer sets were designed for the detection of the APV1 virus (Supplementary Table S5). PCR conditions included 30 cycles of denaturation at 95°C for 15 s, primer annealing at 60°C for 20 s, and extension at 72°C for 1 min (for a 1 kb product), followed by a final extension at 72°C for 5 min, using the SanTaq Plus PCR Mix (Sangon Biotech, China).

## 3 Results

### 3.1 Expression and purification of the recombinant APV1-CP protein

**Figure 1:**
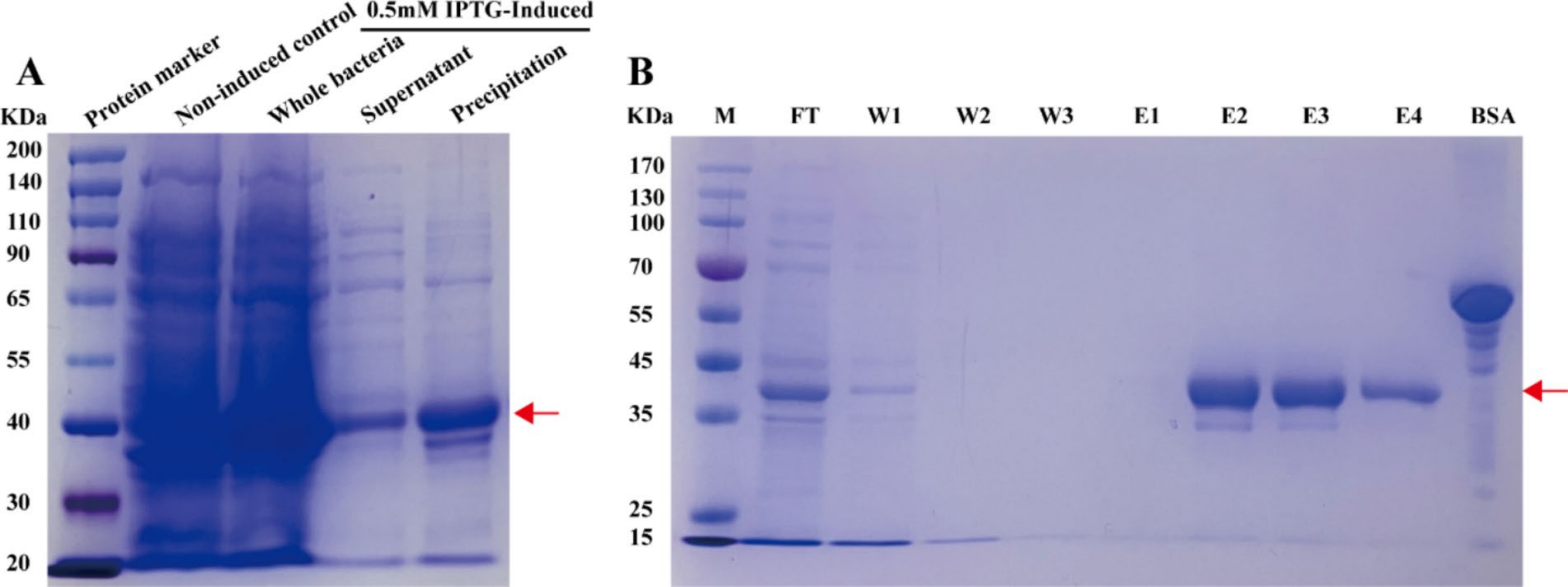
Induction and purification of APV1-CP protein. **(A)** The expression of APV1-CP was determined via sodium dodecyl sulfate-polyacrylamide gel (SDS-PAGE) analysis. **(B)** The purification of APV1-CP was also determined via SDS-PAGE. M, marker; FT, flow through; W1–W3, washing buffer 1–3; E1–E4, elution buffer 1–4. The position of APV1-CP protein in the gel was indicated by an arrow. BSA, bovine albumin, 10 mg/mL.

To obtain soluble APV1-CP protein, we used the *E. coli* expression strain ArcticExpress BL21 (DE3) transformed with a plasmid carrying the His-CP gene. The protein was induced under 0.5 mM ITPG, and SDS-PAGE confirmed successful expression of the 6 × His-tagged recombinant APV1-CP protein (~40-KD) (Figure 1). Ultimately, we obtained over 2.5 mg (0.5 mg/mL in a 1 mL volume) of the recombinant APV1-CP protein for use in further immunization studies.

### 3.2 Preparation of MAbs against APV1-CP

**Figure 2:**
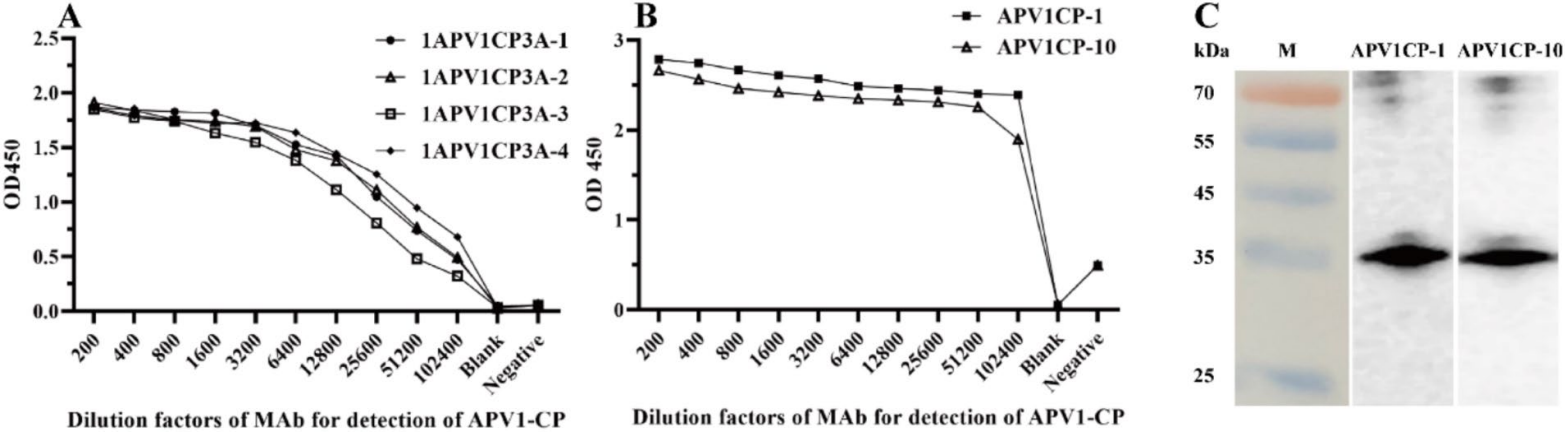
Titer and specificity analysis of APV1 monoclonal antibody. **(A)** The titer of monoclonal antibody serum to APV1-CP recombinant protein was determined by indirect ELISA. **(B)** The titer of purified monoclonal antibody against APV1-CP recombinant protein was used to determine by indirect ELISA. **(C)** Analysis of the specificity of MAb.

Blood samples were collected from the auricular veins of mice at 57 days post-immunization (DPI) for titer evaluation using the indirect ELISA method. After the fifth immunization, antiserum from one of the four mice (1APV1CP3A-4) exhibited the highest titer (1:51,200) against the APV1-CP protein (Figure 2A and Supplementary Table S2).

Spleen cells from the 1APV1CP3A-4 mouse were fused with SP2/0 myeloma cells and plated onto six 96-well cell culture plates. After several rounds of subcloning and screening, nine hybridoma lines 1, 4, 5, 7, 9, 10, 11, 14 and 23 were obtained (Supplementary Table S1). Eventually, hybridoma lines 1 and 10 were selected for MAbs purification, resulting in antibodies APV1CP-1 (2.2 mg/mL) and APV1CP-10 (1.7 mg/mL). The titers of APV1CP-1 and APV1CP-10, as measured by indirect Elisa, exceeded 102,400 (Figure 2B and Supplementary Table S3). Analysis of serial dilutions of the APV1CP-1 and APV1CP-10 antibodies demonstrated that these antibodies possess sufficient sensitivity to detect the APV1-CP. This provides a foundation for further research and development of the CGICS Western blot assay was performed to assess the specificity of the MAbs against the immune antigen APV1-CP. The results indicated that only the APV1-CP protein was detected in the APV1CP-1 and APV1CP-10 (Figure 2C), confirming the high specificity of these MAbs.

### 3.3 Preparation of colloidal gold-labeled MAbs

**Figure 3:**
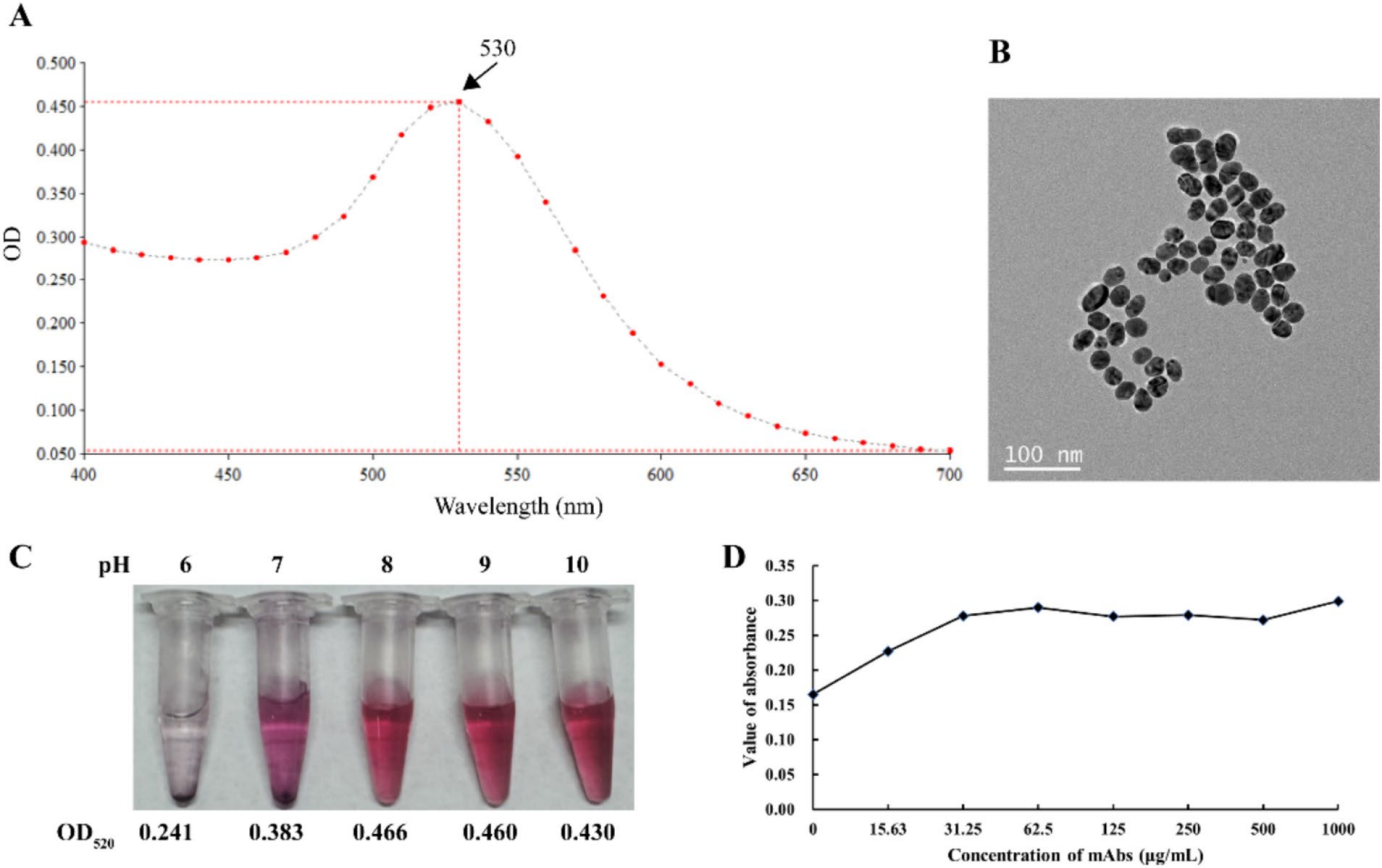
Preparation of colloidal gold-labeled MAbs. **(A)** UV/Vis spectrum of the solution containing the colloidal gold particles. **(B)** TEM image of the particles. **(C)** The optimal pH of APV1CP-1 conjugated with colloidal gold. **(D)** Determining the optimal ratio of colloidal gold and MAb for conjugation.

The colloidal gold solution displayed a characteristic deep red color and high optical clarity. As shown in Figure 3A, the UV/Vis spectrum of colloidal gold demonstrated a peak at approximately 530 nm, corresponding to the surface plasmon resonance of the gold nanoparticles. Transmission electron microscopy (TEM) images further confirmed that the colloidal gold particles were nearly uniform, with diameters ranging from 30 to 40 nm, an optimal range for preparing CGICS (Figure 3B). Figure 3C illustrates that the optimal pH for APV1CP-1 to effectively stabilize colloidal gold and prevent aggregation is pH 8, 9, 10. Therefore, a colloidal gold solution with a pH of 8 was selected to label the antibody (Figure 3C).

To conjugate APV1CP-1 with colloidal gold, the optimal antibody-to-gold ratio was determined using a salt precipitation assay. Colloidal gold (125 μL) was mixed with increasing concentrations of APV1CP-1, ranging from 0 to 1,000 μg/mL. With rising antibody concentrations, the solution’s color transitioned to a deep purple, reaching a saturation point at 31.25 μg/mL (30 μL), beyond which no further color change was observed (Supplementary Figure S1). UV absorbance measurements at OD_528nm_ showed a steady increase in absorbance as the antibody concentration increased from 0 to 31.25 μg/mL, after which the absorbance curve plateaued, remaining nearly constant between 31.25 and 500 μg/mL (Figure 3D). These results indicate that 30 μL of 31.25 μg/mL antibody is the optimal concentration for coupling with 125 μL of colloidal gold.

### 3.4 Assembly of CGICS for detection of APV1

To identify the optimal type of nitrocellulose membrane for assembling the CGICS, we evaluated different brands, including Sartorius 95, Sartorius 140, Huamaike 140, and Whatman 140, for sensitivity and performance. The Huamaike 140 nitrocellulose membrane was selected as the most suitable for the detection of APV1 (data not shown).

**Figure 4:**
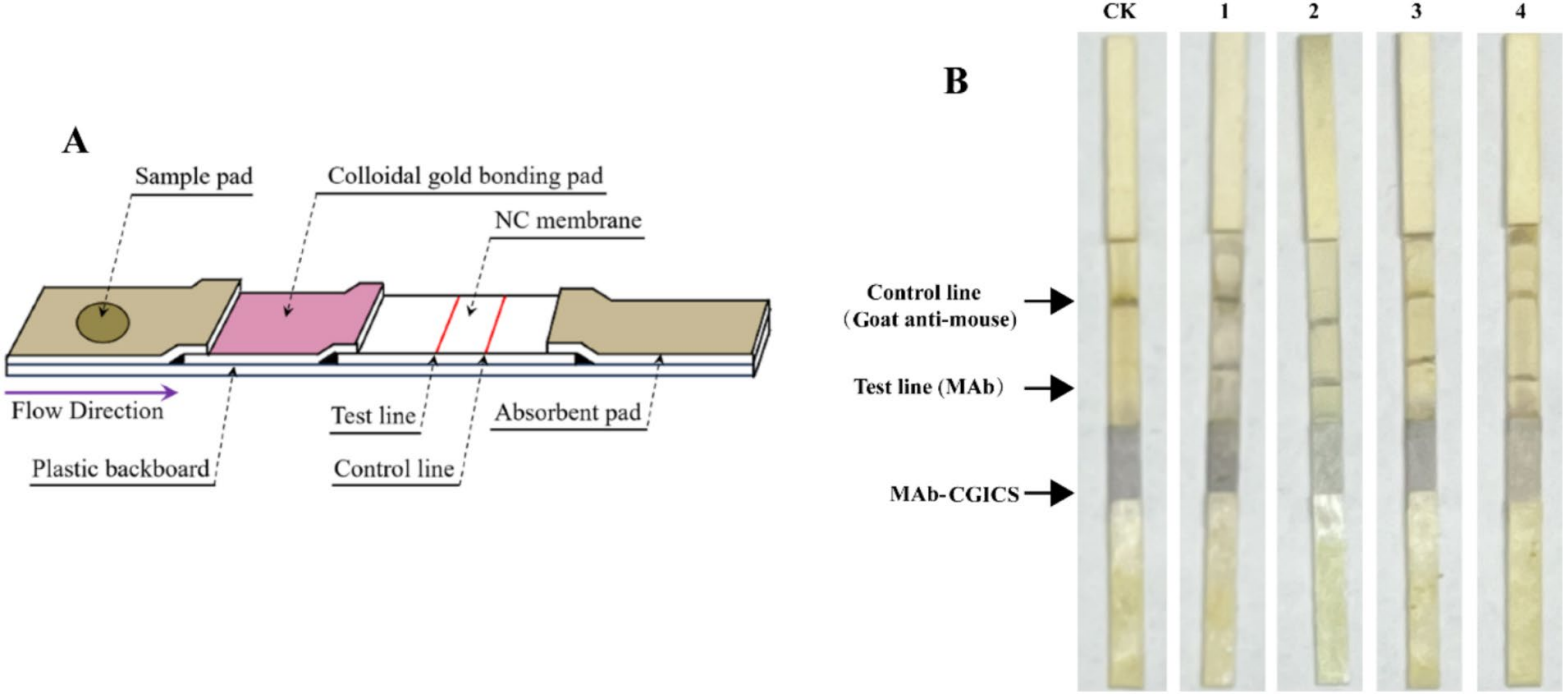
Composition of the colloidal gold immunochromatographic strip for APV1 detection. **(A)** The schematic representation of the APV1-CGICS. **(B)** CGICS were used to crude extract of leaves infected with APV1. CK, leaf extract from asymptomatic plant.

The strips were assembled by sequentially layering a sample absorbing pad, an application pad with conjugated MAb APV1CP-1-colloidal gold, a Huamaike 140 nitrocellulose membrane, and a water-absorbing pad (Figure 4A). To capture the APV1-bound monoclonal antibody APV1CP-1-colloidal gold complexes, another MAb APV1CP-10 was coated at the test line on the nitrocellulose membrane. To ensure that antibody-conjugated colloidal gold particles flowed along the strip, Goat anti-mouse antibody was coated at the control line on the nitrocellulose membrane to capture all antibody-conjugated colloidal gold.

When leaf extracts from APV1-infected and-uninfected plants were separately added to the sample pad, the test line on the strip treated with the APV1-infected leaf extract turned distinctly purple within 5–10 min. In contrast, the test line on the strip treated with the APV1-uninfected leaf extract showed no color change (Figure 4B).

### 3.5 Specificity and sensitivity of the APV1-CGICS

**Figure 5:**
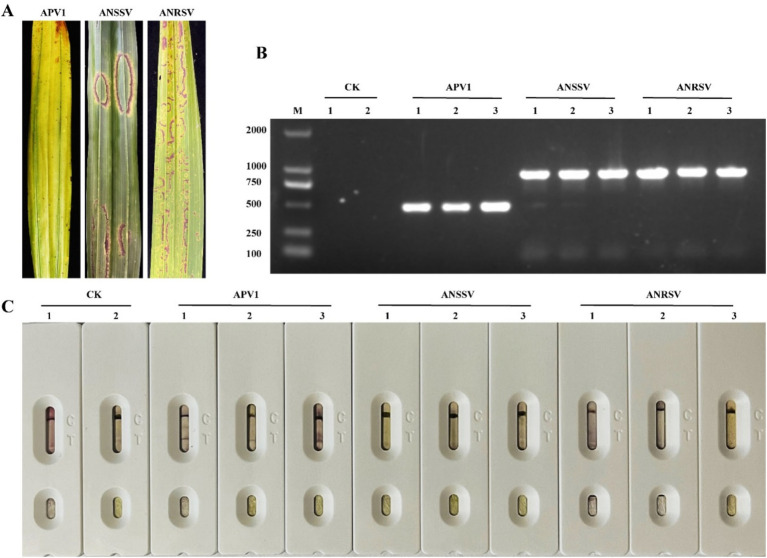
Specificity analysis of the developed colloidal gold immunechromatographic strip. **(A)** The leaf symptoms of areca palm infection with APV1, ANRSV, or ANSSV. **(B)** RT-PCR detection of areca palm samples infected with APV1, ANRSV, and ANSSV, respectively. **(C)** CGICS detection of areca palm samples infected with APV1, ANRSV, and ANSSV, respectively.

To assess the specificity of the APV1-CGICS, we tested extracts from areca palm leaves infected with APV1, ANRSV, and ANSSV. Positive results were observed exclusively in strips treated with APV1-infected samples, with no positive outcomes in strips treated with extracts from any other viruses, consistent with RT-PCR results (Figure 5). Apart from that, we also used the prepared APV1-CP colloidal gold immunochromatographic test strip to detect the *Tobacco rattle virus* (TRV), *Potato virus X* (PVX), and *Rubber Tree Virus 1* (RTV1) stored in our laboratory. The results showed that the test strip specifically recognizes only the APV1 virus (Supplementary Figure S2).

**Figure 6:**
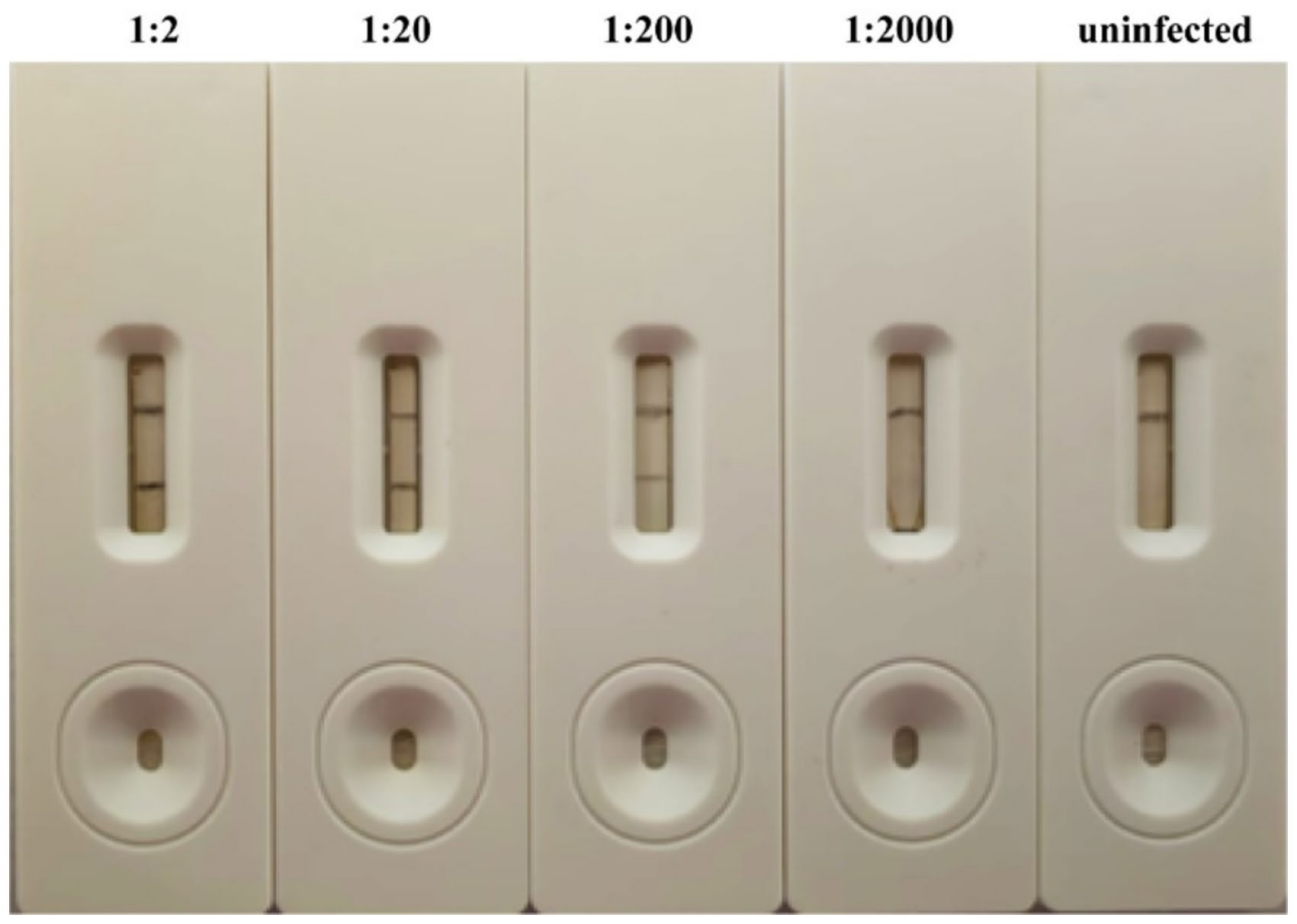
Sensitivity of CGICS for the detection of APV1. A series of diluted leaf extracts from APV1-infected tissue were tested by CGICS. Leaf extract from asymptomatic plant was used as the negative control.

For sensitivity testing, crude extracts from an APV1-infected areca palm leaf were serially diluted in 0.01 mol/L PBS (dilutions from 1:20–1:2,000), and 200 μL of each dilution was applied to the sample pad of the strip, with uninfected leaf extract serving as the negative control. The results demonstrated that the CGICS successfully detected APV1 infection in diluted infected crude extracts up to 1:200 (w/v) dilution (Figure 6), establishing the strip’s detection limit.

Additionally, we compared the performance of the monoclonal antibody (MAb)-based CGICS with that of a batch of rabbit polyclonal antibody (PAb)-based CGICS. The results indicated a significantly higher sensitivity of the MAb-CGICS compared to the PAb-CGICS (Supplementary Figure S3).

### 3.6 Controlled test and field application of the APV1-CGICS

**Figure 7:**
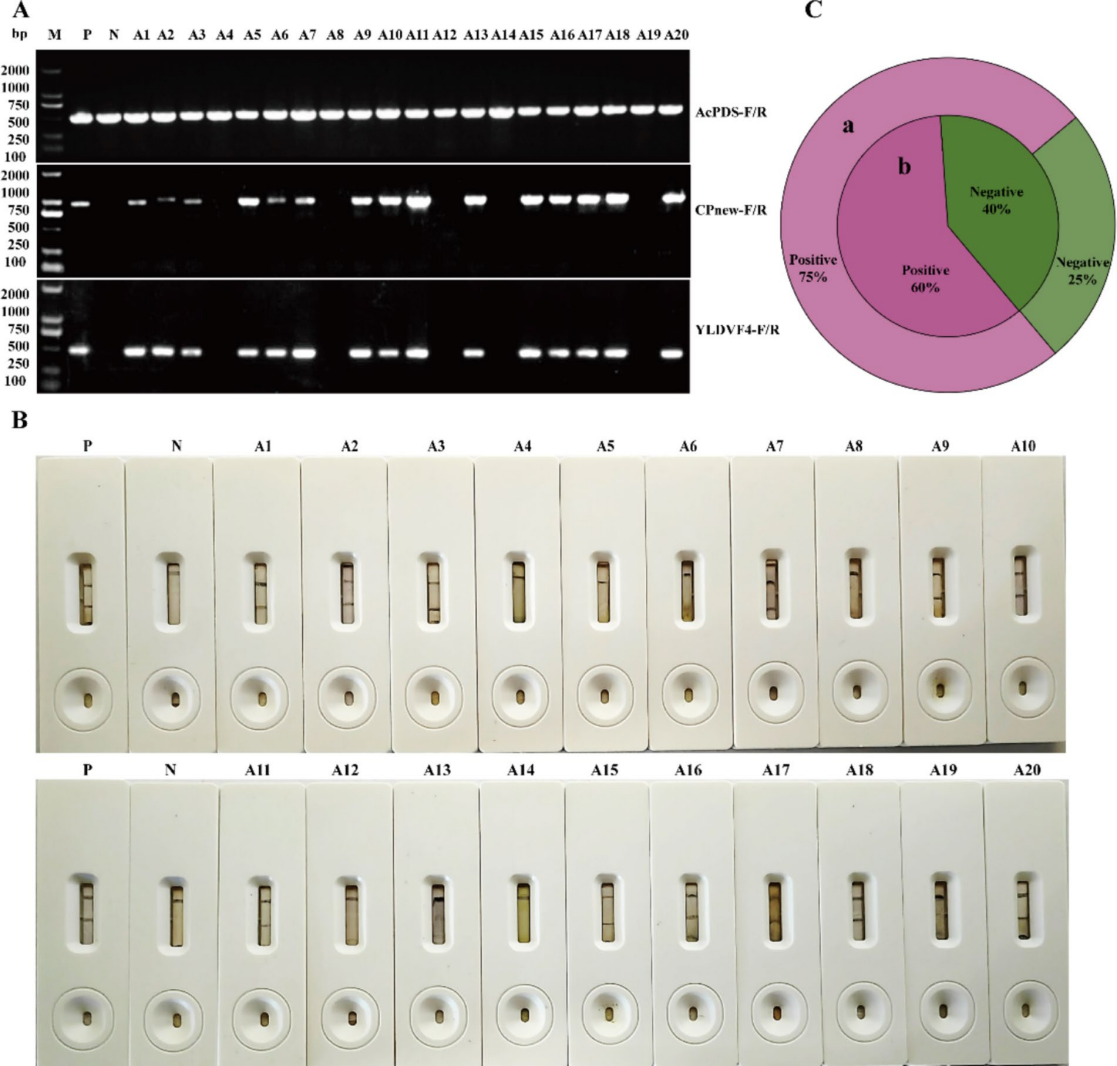
Field application of the APV1-CGICS. **(A)** Detection of APV1 in field samples via reverse-transcription PCR, M, DNA size 2000 marker; P, positive control; N, negative control. CK-1 and CK-2, asymptomatic areca palm. **(B)** Detection of APV1 in field samples using the APV1-CGICS. **(C)** The detection rate of APV1. **(a)** Use RT-PCR for detection. **(b)** Use APV1-CGICS for detection.

To determine the effectiveness of the newly developed APV1-CGICS for detecting APV1, we tested 20 field samples from areca palms (A1–A20) exhibiting the YLD symptoms. Of these, 12 samples (A1, A2, A3, A7, A9, A10, A11, A15, A16, A17, A18, and A20) were tested positive, while 8 samples were negative, resulting in a detection rate of 60% (Figures 7B,C). RT-PCR was performed on all the 20 samples to verify the presence of APV1. RT-PCR results confirmed that 15samples were positive (Figure 7A), resulting in a detection rate of 75% (Figure 7C), indicating an agreement rate of 85% between CGICS and RT-PCR. These findings collectively suggest that the APV1-CGICS can be effective for the rapid and precise detection of APV1 in field conditions.

## 4 Discussion

APV1 is the most devastating pathogen affecting areca palms, with a detection rate exceeding 70% in areas experiencing YLD in Hainan Province, China (Wang et al., 2020; Niu et al., 2021; Chen et al., 2022). Research has shown that APV1 often invades new geographical areas by introducing infected areca palms seedlings. Early detection and rapid elimination of infected seedlings through strict inspection and quarantine can effectively block this transmission chain. Therefore, establishing a simple and reliable APV1 detection technology is of great significance for the prevention and control of YLD. Such as RT-PCR, real-time PCR, and ELISA are widely used for APV1 virus detection, but they require expensive instruments and equipment, and the operation is complex, time-consuming, and labor-intensive, unsuitable for rapid on farms and customs sites (Cao et al., 2021; Lin et al., 2021; Chen et al., 2022). Colloidal gold immunochromatographic strip (CGICS) is a fast, simple, economical and practical on-site serological testing technology, especially suitable for large-scale field investigations and inspection and quarantine at customs ports. At present, there is no CGICS for detecting APV1.

High sensitivity and specificity antibodies are crucial for developing effective serological detection methods. In this study, purified recombinant APV1-CP protein was obtained and used to develop specific MAbs APV1CP-1 and APV1CP-10. Both MAbs can specifically react with APV1-CP, the titers of APV1CP-1 and APV1CP-10, as measured by indirect Elisa, exceeded 102,400 (Figure 2 and Supplementary Table S3). After cross-combination analysis of the two antibodies, we determined that APV1CP-1 as the labeled antibody and APV1CP-10 as the trapping antibody can obtain higher accuracy and sensitivity. The reliability of the CGICS results is influenced by several factors, with the quality of colloidal gold particles—specifically their uniformity and particle size—playing a key role in the preparation of CGICS (Zhu et al., 2022). Colloidal gold particles typically have a diameter ranging from 5 to 150 nm, but for diagnostic assays, particles in the range of 20 to 40 nm are generally preferred. Smaller colloidal gold particles facilitate better mixing on the adsorption line, thereby enhancing detection sensitivity (Li et al., 2019; Zhu et al., 2022). In this study, dispersed colloidal gold particles with a uniform diameter of 40 nm were prepared using a 1:1 ratio of hydrogen tetra-chloroaurate hydrate to trisodium citrate solution, which improves the stability and flow characteristics of the colloidal gold on the membrane (Figures 3A,B). Furthermore, the optimal pH for colloidal gold-antibody binding was determined to be 8 (Figure 3C).

We developed a CGICS using MAbs for rapid detection of APV1. This method allows for analysis within 5–10 min, we have developed a highly specific and sensitive APV1 detection strip using these two MAbs. This test strip can be used within 5–15 min, APV1 was detected sensitively in areca palm leaf tissues, with a sensitivity of 1:200 (g/mL) dilution for crude extract of leaf tissue. The detection of the other two analyzed viruses (ANRSV, ANSSV) and uninfected plant tissue showed negative reactions (Figures 5, 6).

In this study, the MAb-CGICS demonstrated significantly higher sensitivity compared to the PAb-CGICS. The CGICS maintained a positive signal with up to a 200-fold dilution (w/v) of the APV1-infected leaf sample from the areca palm, highlighting its superior sensitivity (Figure 6; Supplementary Figure S3). For comparison, the visual detection limit of the test strip for SMV, TZSV, and Plum pox virus (PPV) was 800-fold, 1,000-fold, and 6,400-fold dilutions of infected leaf samples, respectively (Niu et al., 2018; Ren et al., 2022; Guo et al., 2023). Those virus titers in leaf tissues infected with viruses from the genera *Potyvirus* (SMV) and *Tospovirus* (TZSV) were generally high (Kogovšek et al., 2011; Schneider et al., 2011; Zhang et al., 2016). In contrast, APV1, a member of the family *Closteroviridae*, primarily infects and replicates in the phloem tissue of plants but can also invade mesophyll cells later in the infection process (Medina et al., 1999; Folimonova et al., 2008; Sun and Folimonova, 2019; Cao et al., 2024). Consequently, the minimum dilution of APV1-CGICS samples is lower than that of samples infected by other viruses. In addition, the waxy layer on the surface of the areca palm leaf can hinder complete grinding and virus release. Despite these challenges, the CGICS developed in this study effectively meets the detection requirements.

CGICS represents a rapid, user-friendly, and cost-effective serological approach, making it especially apt for large-scale field investigations (Dong et al., 2022; Guo et al., 2023). In this study, an in-depth analysis of 20 collected samples furnished further corroboration of the effectiveness and precision of the APV1-GICS technology in detecting APV1 (Figure 7). Notably, it exhibited an 85% concordance rate with RT-PCR outcomes (Figure 7), underlining its reliability. Phylogenetic analysis divided the APV1 isolates into three phylogroups, with a preponderant 16 isolates (>70%) clustering within phylogroup A (Dong et al., 2022; Guo et al., 2023). Based on these findings, we postulate that the APV1 test strip devised in this paper has the potential to detect APV1-A or other related APV1 strains. Meanwhile, it was surmised that impurities and pigments present in the crude extract might impede the liberation of the colloidal gold-APV1CP-1 conjugates and compromise the antibody binding efficiency. Moreover, the titer of APV1 was suspected to be lower in the leaf tissue from severely yellowed areca palms. Looking ahead, future endeavors will be centered around optimizing CGICS This will entail refining the sample grinding protocol to enhance homogenization and implementing more efficient filtration methods for crude extracts,. all with the overarching goal of augmenting the detection proficiency of CGICS in field conditions. During large-scale production, ensuring the long-term efficacy of antibodies is of paramount importance. Specifically, it is necessary to maintain antibody functionality over an extended timespan (e.g., within 1 month). Therefore, we will embark on exploring the incorporation of diverse preservatives during the preparation of the test strips to prolong the validity of their detection results. Furthermore, due to pronounced seasonal temperature fluctuations in the field, we are poised to conduct studies on the stability and performance of the test strips under varying temperature regimens, thereby guaranteeing their reliability and applicability across different environmental conditions.

In conclusion, we have successfully developed two highly sensitive and specific MAbs and utilized them to create an ultra-sensitive, reliable, and user-friendly CGICS for detecting APV1. We strongly advocate for the adoption of this method in APV1 field surveys, certification of APV1-free areca palm materials, and phytosanitary inspections.

## Data Availability

The original contributions presented in the study are included in the article/Supplementary material, further inquiries can be directed to the corresponding authors.
