# Supplementary material for: Development of a colloidal gold-based immunochromatographic strip for rapid detection of Areca palm velarivirus 1
Source: Front Microbiol. 2025 Feb 20;16:1533170. doi: 10.3389/fmicb.2025.1533170 (PMC11892987; doi:10.3389/fmicb.2025.1533170)
Supplement: Supplementary file 1 [file Data_Sheet_1.docx]

Supplemental materials


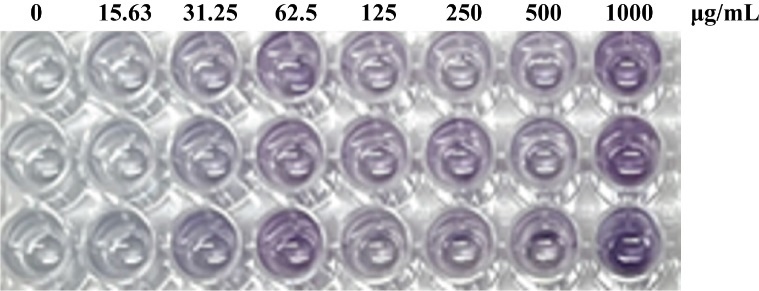


**Figure S1 A fixed amount of colloidal gold (200 μL) was mixed with an increasing amount, from 0 to 1 mg/mL (30 μL), of mAb APV1CP-1.** As the concentration of the antibodies increased, the color of the conjugated antibody-colloidal gold became purple.


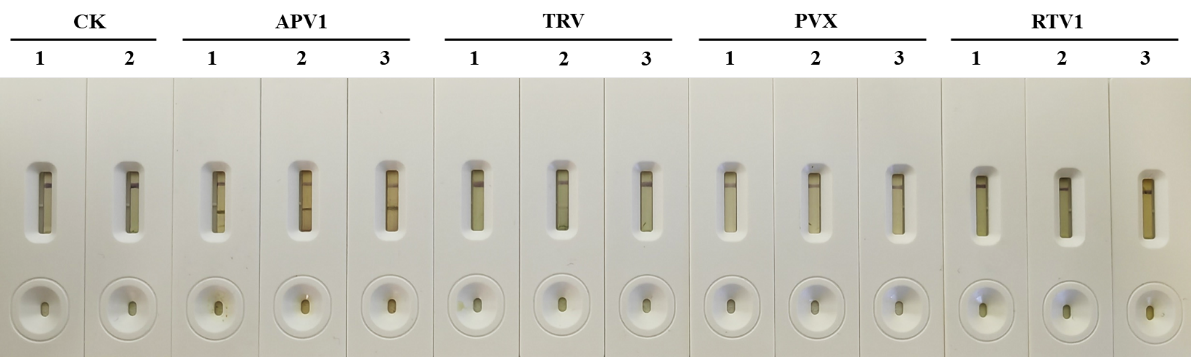


**Figure S2. CGICS detection of samples infected with TRV, PVX, and RTV1, respectively.** CK, Leaf extract from Asymptomatic *Nicotiana benthamiana*; APV1, *Areca palm velarivirus 1*; TRV, *Tobacco rattle virus*; PVX, *Potato virus X*; RTV1, *Rubber Tree Virus 1*.


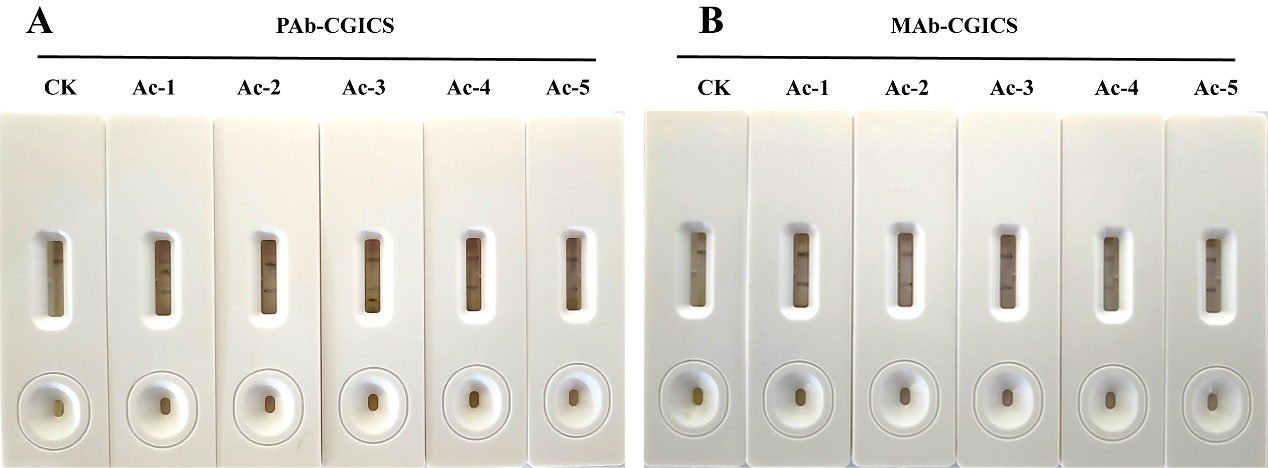


**Figure S3. Comparative analysis of PAb-CGICS and MAb-CGICS.** A: Detection of APV1 using the PAb-CGICS; B: Detection of APV1 using the MAb-CGICS.

**Table S1. Information of 9 positive hybridoma cell lines**

| number | hybridoma lines | OD value for immunogen | OD values for screening proteins | subtype |
| --- | --- | --- | --- | --- |
| 1 | 1 | 0.681 | 0.122 | G1 |
| 2 | 4 | 0.645 | 0.042 | G1 |
| 3 | 5 | 0.638 | 0.041 | G1 |
| 4 | 7 | 0.647 | 0.041 | G1 |
| 5 | 9 | 0.679 | 0.054 | G1 |
| 6 | 10 | 0.764 | 0.059 | G1 |
| 7 | 11 | 0.665 | 0.06 | G1 |
| 8 | 14 | 0.67 | 0.033 | G2a |
| 9 | 23 | 0.637 | 0.04 | G1 |
| negative |  | 0.001 | 0.019 |  |
| blank |  | 0.015 | 0.023 |  |
| positive |  | 0.763 | 0.646 |  |

**Table S2. Detection of antibody titer in immunized mouse serum**

| Dilutions | 200 | 400 | 800 | 1600 | 3200 | 6400 | 12800 | 25600 | 51200 | 102400 | Blank | Negetive |
| --- | --- | --- | --- | --- | --- | --- | --- | --- | --- | --- | --- | --- |
| 1APV1CP3A-1 | 1.868 | 1.842 | 1.828 | 1.813 | 1.7 | 1.525 | 1.427 | 1.048 | 0.739 | 0.474 | 0.049 | 0.053 |
| 1APV1CP3A-2 | 1.912 | 1.839 | 1.755 | 1.74 | 1.693 | 1.48 | 1.379 | 1.111 | 0.768 | 0.493 | 0.028 | 0.06 |
| 1APV1CP3A-3 | 1.849 | 1.773 | 1.74 | 1.632 | 1.546 | 1.382 | 1.114 | 0.809 | 0.48 | 0.322 | 0.035 | 0.055 |
| 1APV1CP3A-4 | 1.864 | 1.79 | 1.75 | 1.721 | 1.725 | 1.636 | 1.443 | 1.255 | 0.946 | 0.68 | 0.03 | 0.056 |

The titer is the dilution corresponding to the minimum OD reading greater than the maximum OD/2.

**Table S3. Titer determination of purified monoclonal antibodies**

| Name | 200 | 400 | 800 | 1600 | 3200 | 6400 | 12800 | 25600 | 51200 | 102400 | Blank | Negetive |
| --- | --- | --- | --- | --- | --- | --- | --- | --- | --- | --- | --- | --- |
| APV1-3-1 | 2.785 | 2.745 | 2.667 | 2.609 | 2.571 | 2.489 | 2.463 | 2.442 | 2.404 | 2.391 | 0.055 | 0.497 |
| APV1-3-10 | 2.664 | 2.564 | 2.464 | 2.422 | 2.383 | 2.348 | 2.334 | 2.312 | 2.257 | 1.896 | 0.055 | 0.497 |

The titer is the dilution corresponding to the minimum OD reading greater than the maximum OD/2.

**Table S5. List of primers used in this study.**

| Primer name | Sequence (5'-3') | Description |
| --- | --- | --- |
| YLDVF4 | atctggaccgagtaatggga | RT-PCR detection for APV1 |
| YLDVR4 | ACATTGTGATACACATACAAGT |  |
| CPnewF | ATCGCTAAATATTATGGATAGACTT |  |
| CPnewR | TATTCAGAAGCATAAGATTGTGACA |  |
| AcPDS-F | TGCAGAACTTGTTTGGGGAAC | RT-PCR detection for the 15-cis-phytoene  desaturase of the areca palm |
| AcPDS-R | CCACCCAATGACTGAATGT |  |
| ANSSV-F | CAAGGATTTTGCAAACAAGGA | RT-PCR detection for ANSSV |
| ANSSV-R | GAAACTCACTGCCGTACAACC |  |
| ANRSV-F | CACGCAAGTAAGGAATTTCAA | RT-PCR detection for ANRSV |
| ANRSV-R | CATTGAGTTGAAGCTCAATGC |  |
